# Identification of a key water molecule involved in the macrophage migration inhibitory factor‐catalyzed tautomerization of *para*‐hydroxyphenylpyruvate using neutron crystallography

**DOI:** 10.1002/pro.70666

**Published:** 2026-08-08

**Authors:** Gabriela C. Schröder, Gregg V. Crichlow, Ewelina Jablonowski, Georgios Pantouris, Jay Nix, Naomi Chayen, Flora Meilleur, Elias J. Lolis

**Affiliations:** ^1^ Department of Molecular and Structural Biochemistry North Carolina State University Raleigh North Carolina USA; ^2^ Neutron Scattering Division Oak Ridge National Laboratory Oak Ridge Tennessee USA; ^3^ Department of Pharmacology Yale University School of Medicine New Haven Connecticut USA; ^4^ Molecular Biology Consortium, Advanced Light Source Lawrence Berkeley National Laboratory Berkeley California USA; ^5^ Division of Systems Medicine, Department of Metabolism Digestion and Reproduction, Imperial College London London UK; ^6^ Present address: MAX IV Laboratory Lund University Lund Sweden; ^7^ Present address: Research Collaboratory for Structural Bioinformatics Protein Data Bank Institute for Quantitative Biomedicine Rutgers, The State University of New Jersey Piscataway New Jersey USA; ^8^ Present address: St. George's University, University Centre Grenada West Indies; ^9^ Present address: Department of Chemistry University of the Pacific Stockton California USA

**Keywords:** 3‐(4‐hydroxyphenyl)‐pyruvate, HPP, keto‐enol tautomerization, macrophage migration inhibitory factor, MIF, neutron crystallography

## Abstract

Neutron crystallography was used to determine a 2.5‐Å resolution all‐atom structure of macrophage migration inhibitory factor (MIF) interacting with 3‐(4‐hydroxyphenyl)‐pyruvate (HPP). MIF is a pro‐inflammatory, pro‐tumorigenic protein that may be an attractive therapeutic target. MIF catalyzes the interconversion of the keto and enol forms of HPP by a tautomerase reaction. Although HPP is evidently not a physiological substrate of MIF, many compounds that inhibit this activity in enzymatic assays have been found also to inhibit physiological activities of MIF. Therefore, the MIF‐catalyzed HPP tautomerization reaction is used in initial screening of compounds in the search for inhibitors of MIF physiological activity. The neutron diffraction‐derived crystal structure reveals the position of a water molecule involved in the tautomerization reaction, and also confirms the charged state of lysine‐32 in the active site. The structure confirms the previously proposed catalytic mechanism of MIF, with the N‐terminal Pro‐1 abstracting a proton to generate an HPP enolate intermediate which is subsequently protonated. The structure reported herein reveals that this proton is supplied by a neighboring water molecule. Along with the neutron structure, a room‐temperature synchrotron x‐ray crystal structure reveals a covalent adduct between HPP and MIF. While this adduct is a result of radiation‐induced chemistry, its formation confirms the catalytic role of the active site residue because a covalent complex could only form if the reactive carbon of the substrate is correctly positioned by the enzyme.

## 1 INTRODUCTION

**FIGURE 1:**
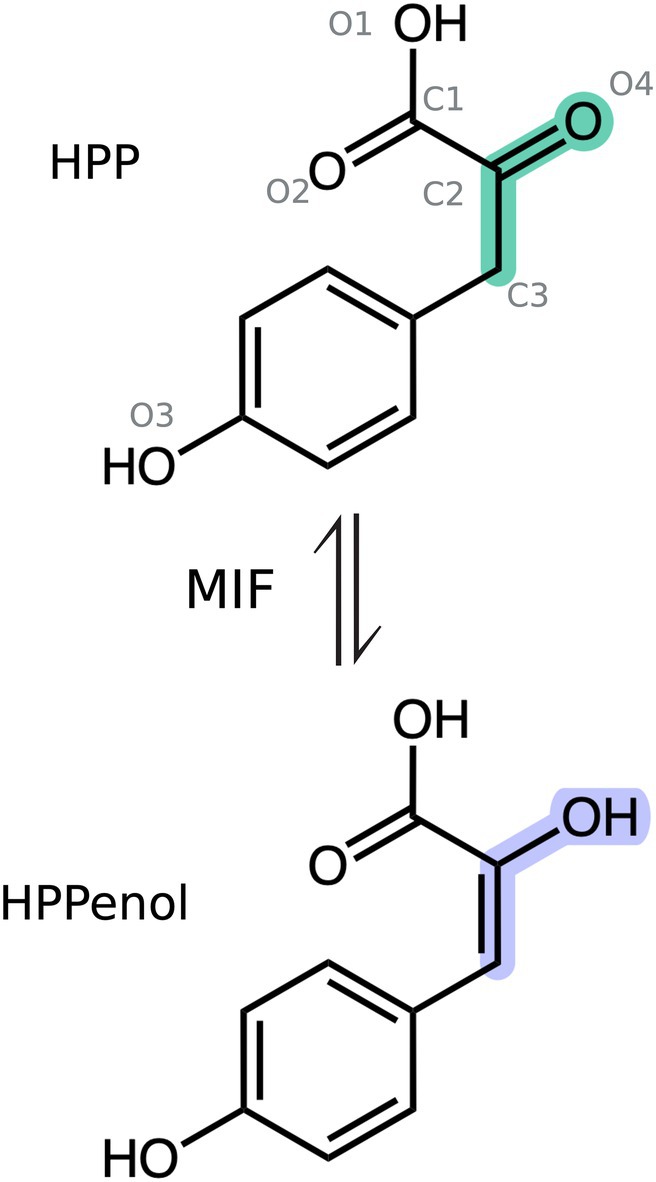
MIF tautomerase reaction. Macrophage migration inhibitory factor (MIF) catalyzes the keto‐enol tautomerization of the ketone 3‐(4‐hydroxyphenyl)‐pyruvate (HPP) to 3‐(4‐hydroxyphenyl)‐enolpyruvate (HPPenol).

Human macrophage migration inhibitory factor (MIF) is a pro‐inflammatory, pro‐tumorigenic cytokine (Calandra et al., 1995; Schrader et al., 2009; Weiser et al., 1989) that is expressed in a variety of tissues (Sumaiya et al., 2022). MIF is expressed as a 114‐residue homotrimer (Kato et al., 1996; Sun et al., 1996), and is known to catalyze the keto‐enol tautomerization of the ketone 3‐(4‐hydroxyphenyl)‐pyruvate (*para*‐hydroxyphenylpyruvate, HPP) to 3‐(4‐hydroxyphenyl)‐enolpyruvate (herein referred to as HPPenol), as well as the reverse reaction (Figure 1) (Johnson Jr. et al., 1999; Rosengren et al., 1997).

**FIGURE 2:**
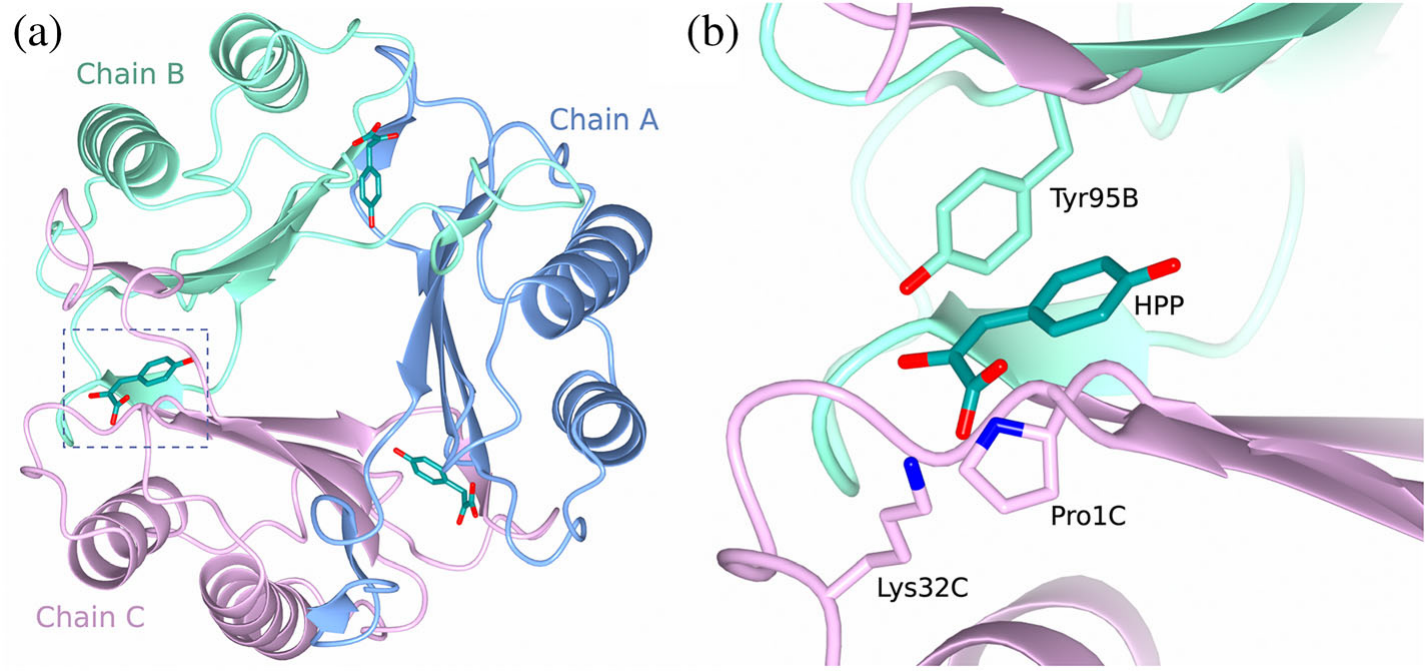
Overall structure of human MIF. (a) MIF forms a trimer composed of three chains with three equivalent tautomerase active sites. (b) The HPP substrate binds to an active site between adjacent subunits in proximity to the Pro‐1 catalytic base.

In an aqueous solution, the keto tautomer is thermodynamically favored, and a mixture of both tautomers spontaneously equilibrates to the keto form in approximately 1 day. The K_M_ of HPP binding to MIF (2.4 mM) is much larger than the measured concentration of HPP in human plasma (0.38 +/− 0.23 μM), and it is assumed that HPP is not a physiological substrate for MIF (Deutsch, 1997; Rosengren et al., 1997). However, the HPP tautomerase enzymatic assay is used to screen for MIF inhibitors that may have an impact on MIF biological functions. A number of compounds that inhibit the MIF tautomerase activity have been found to inhibit the biological functions of MIF as well (Crichlow et al., 2007; Ioannou et al., 2014; Lubetsky et al., 2002). Many of these inhibitors bind to the three equivalent tautomerase active sites located between adjacent subunits of the MIF trimer (Figure 2), where the catalytic base, Pro‐1, is located. Pro‐1 also reacts with dietary isothiocyanates, undergoing an irreversible covalent modification. MIF‐binding of isothiocyanates in the tautomerase active site is supposed to contribute to the cancer‐preventive properties of dietary isothiocyanates (Brown et al., 2009; Crichlow et al., 2012; Cross et al., 2009; Ouertatani‐Sakouhi et al., 2009; Tyndall et al., 2012).

**FIGURE 3:**
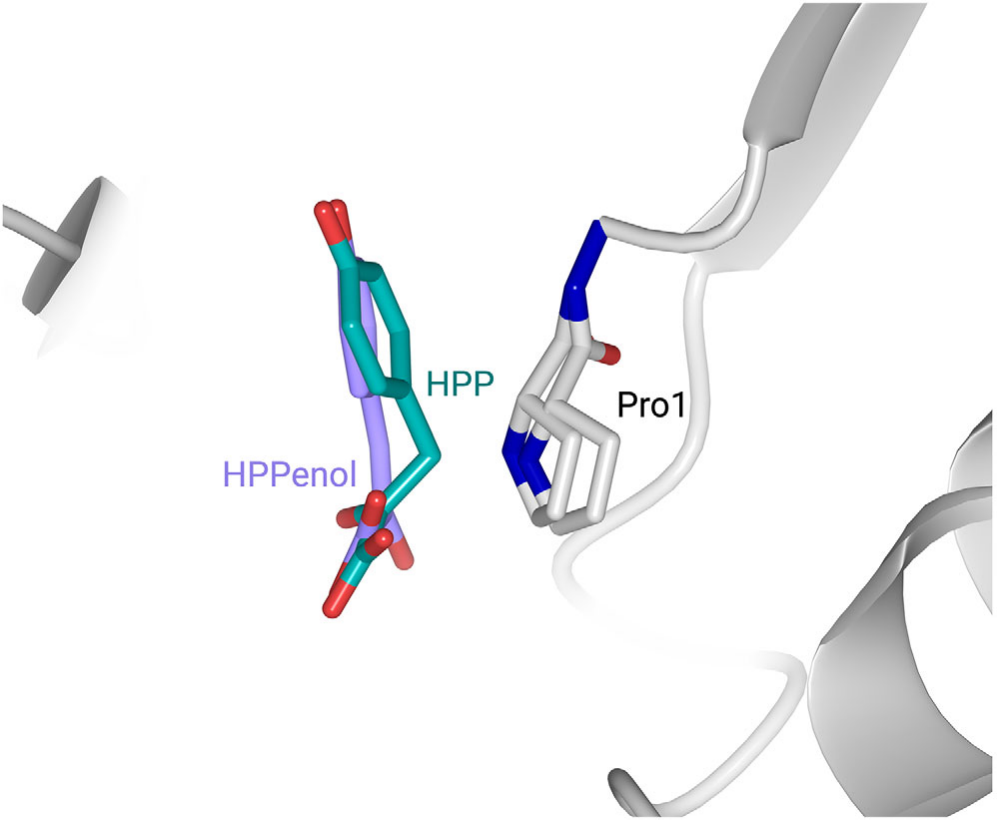
High resolution uninhibited MIF active site. HPP bound to MIF in one of the two uninhibited active sites (active site A) of the 1.25‐Å cryo‐temperature structure (PDB ID 3IJJ, Cho et al., 2010). The added substrate, HPP, is shown proximal to the active site proline, and the enol tautomer product, 3‐(4‐hydroxyphenyl)‐enolpyruvate (HPPenol), is shown in the center of the active site cavity.

A prior 1.25‐Å resolution structure of MIF bound to HPP (PDB ID 3IJJ) shows both the substrate and product (HPP and HPPenol) in the active site, as expected because the two forms can be interconverted by the enzyme (Cho et al., 2010). Interestingly, the enol form was found in a different position in the active site than the keto form of HPP. An earlier 2.5‐Å crystal structure of MIF bound to HPP (PDB ID 1CA7) showed the ligand centrally located in the active site (Lubetsky et al., 1999). Although the keto and enol forms cannot be distinguished at this resolution, HPPenol in the *E*‐configuration was modeled in the active site to best fit the electron density. The 1.25‐Å structure provided sufficient resolution to resolve the enol and keto species in the active site. It also confirmed that it is the enol form that is centrally located in the active site, based on bond angle considerations (Cho et al., 2010). The data supported modeling of the keto form of HPP in an overlapping region, positioned closer to Pro‐1. The dihedral angles of the Pro‐1 proximal species are consistent with the keto form and ruled out the enol form, in which the geometry must be either *E* or *Z* in the C2‐C3 bond, confirming assignment of keto HPP in this portion of the active site. In the previous high‐resolution structure, one of the three active sites has a co‐crystallized allosteric inhibitor bound proximal to it. This led to a supposed intermediate being observed instead of the keto form of HPP, which is in a slightly different position and conformation relative to the keto form of HPP. However, the position of the keto form in the two uninhibited active sites is essentially identical. In the uninhibited active sites, the C3 atom of the keto form of HPP was found to be proximal to the nitrogen atom of Pro‐1 (Figure 3) (Cho et al., 2010). This suggests a mechanism in which the Pro‐1 nitrogen abstracts a proton from C3 of HPP, as proposed by Lubetsky et al. (1999), which is made possible by the singly‐protonated state of Pro‐1 in unbound MIF at physiological pH (Swope et al., 1998). However, hydrogen atoms cannot be visualized by x‐ray diffraction except at very high resolution. We therefore determined the crystal structure of MIF bound to HPP using neutron diffraction, and report the results herein.

## 2 RESULTS

### 2.1 Modeling of the active site ligands

The crystal structure of MIF bound to HPP was determined by neutron diffraction at a resolution of 2.53 Å to visualize hydrogen atoms involved in the tautomerase reaction. To determine the hydrogen atom positions from the neutron scattering length density (NSLD) maps, the HPP was deuterated at carbon C3 before adding it to the enzyme, and the co‐crystal was exposed to heavy water vapor for hydrogen/deuterium exchange of the labile hydrogen atoms. Inspection of the NSLD maps of the 2.53 Å dataset confirmed that the substrate was bound in all three active sites. For all three MIF chains, the active site NSLD spanned the Pro‐1‐proximal area and the substrate binding region of the active site. Due to the limited resolution, any alternate substrate binding conformations were not modeled. HPP tautomers were modeled in the three active sites of the MIF trimer, and the electron and NSL densities were analyzed to determine the predominant species bound in each active site. The ligand was modeled as either keto or enol based on the hydrogenation state of Pro‐1 and on which assignment yielded better refinement statistics, although partial occupancy of both forms is likely present in each active site. The ligands all refined to optimum positions located near the center of the active site pocket, consistent with the HPPenol reported in PDB entries 3IJJ (Cho et al., 2010) and 1CA7 (Lubetsky et al., 1999).

### 2.2 HPP‐MIF interactions

**FIGURE 4:**
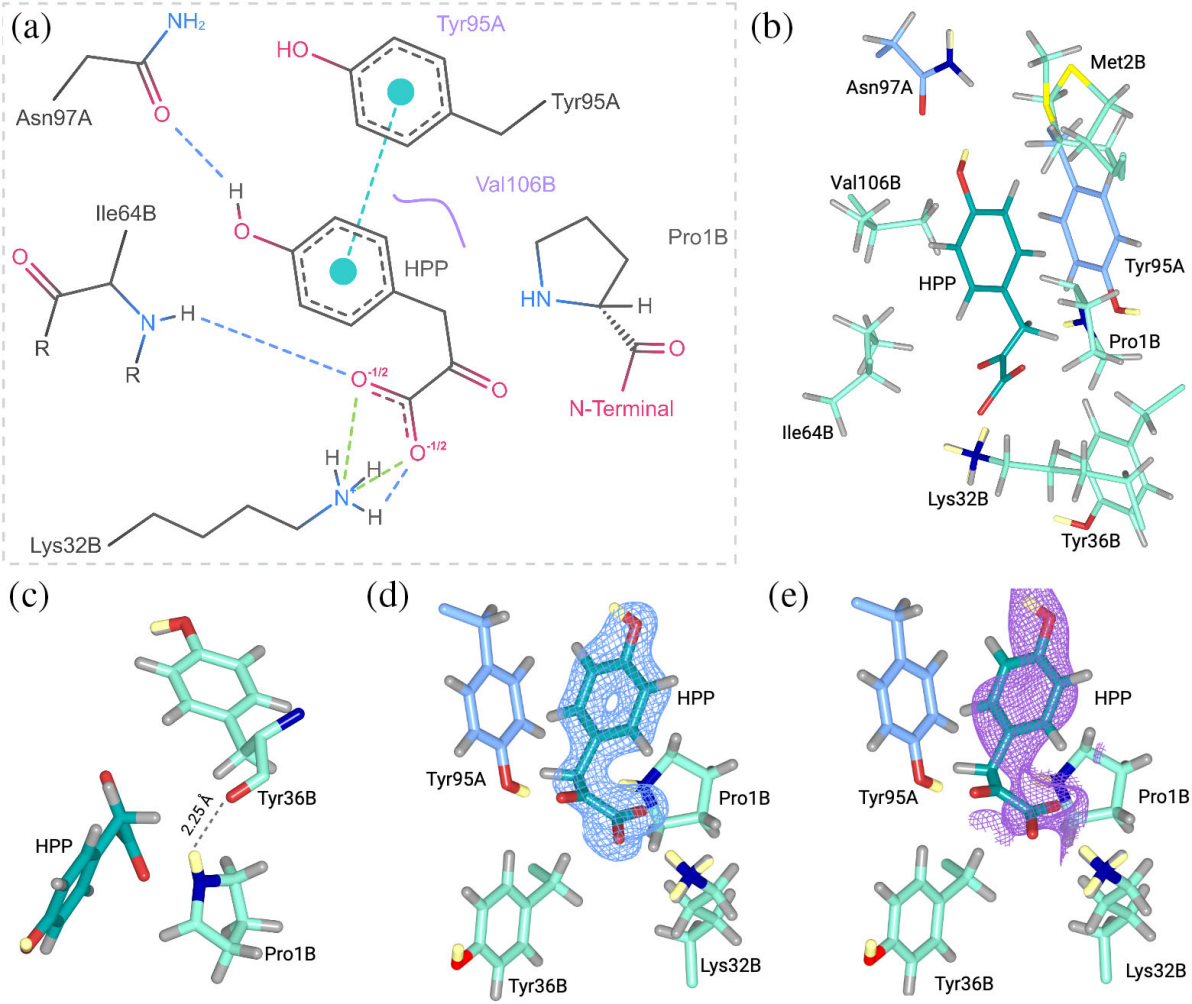
Active site environment in HPP‐bound MIF. (a, b) Interactions of active site residues with HPP bound to chain B of MIF. Tyr‐95A and Asn‐97A are from an adjacent subunit of the trimer. The wavy purple line in (a) indicates a hydrophobic contact. (c) The active site Pro‐1 nitrogen donates a hydrogen bond to the nearby Tyr‐36 main chain carbonyl oxygen. (d) The 2F_o_‐F_c_ electron density map (blue) of HPP bound to chain B of MIF in the joint x‐ray/neutron refined structure (PDB ID 8TT8), contoured at 1.0σ. (e) The 2F_o_‐F_c_ neutron scattering length density map (purple) of HPP bound to chain B of MIF in the joint x‐ray/neutron refined structure, contoured at 1.5σ with deuterium atoms in yellow and hydrogen atoms in gray.

**FIGURE 5:**
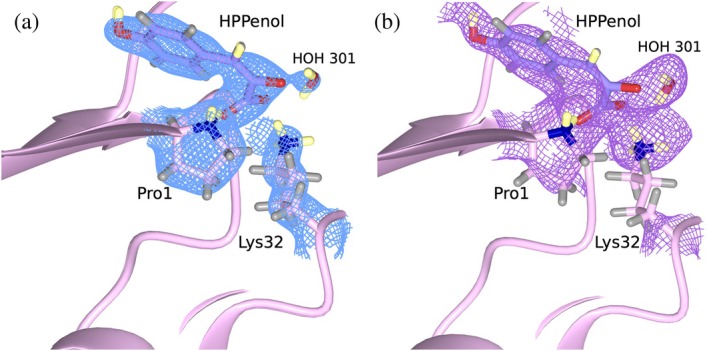
HPPenol bound to active site C in the joint x‐ray/neutron refined structure of MIF‐HPP (PDB ID 8TT8). (a) The 2F_o_‐F_c_ electron density map (blue) of HPPenol bound to active site C, contoured at 0.90σ. (b) The 2F_o_‐F_c_ neutron scattering length density map (purple) of HPPenol bound to active site C, contoured at 1.0σ with deuterium atoms in yellow and hydrogen atoms in gray.

The carboxylate group of HPP was found to participate in electrostatic interactions with the positively charged Lys‐32 side chain amino group, in agreement with previously determined crystal structures (Cho et al., 2010; Lubetsky et al., 1999). The carboxyl group is positioned midway between the Lys‐32 amino group and the Pro‐1 nitrogen, with each oxygen atom located within 2.6 Å of the respective nitrogen atoms, as indicated by refinement results. The oxygen atoms of the HPP carboxyl group are partially directed toward the Lys‐32 ε‐amino group. The edge of the HPP aromatic ring interacts with the π‐face of the aromatic ring of Tyr‐95 of a neighboring chain within the trimer and the active site pocket is lined predominantly with hydrophobic residues (Figure 4a,b). The phenol hydroxyl group of the substrate forms a hydrogen bond to the side chain oxygen of Asn‐97 of a neighboring chain within the trimer. The interaction between the HPP phenolic hydroxyl group and the Asn‐97 side chain has also been reported in earlier MIF crystal structures. The NSLD maps indicate that the hydrogen/deuterium atom on the Pro‐1 nitrogen extends toward the carbonyl oxygen of Tyr‐36, donating a hydrogen bond (Figure 4c). Joint x‐ray/neutron refinement allows determination of the HPP and Pro‐1 hydrogen/deuterium atom positions, allowing us to identify the charged state of Pro‐1 in each active site. Due to the likely occurrence of both substrate and product at partial occupancies, there is expected to be partial occupancy of a second deuterium atom bound to the Pro‐1 nitrogen. However, if the keto form of the substrate is the predominant form in the active site, the Pro‐1 nitrogen will be primarily singly deuterated. The occupancy of the other deuterium would be too low for it to be observable in the NLSD map. Therefore, in active sites A and B, HPP was modeled as the keto form based on the singly deuterated Pro‐1 residues, and on improved refinement statistics (Figure 4d,e), although the resolution is neither sufficient to model both tautomers of HPP with partial occupancies nor to determine the identity of a specific tautomer unambiguously. In active site C, a doubly deuterated Pro‐1 was observed, and therefore we modeled HPPenol in active site C, which occupies its previously found location in the central portion of the active site cavity (Figure 5). The NSLD 2F_o_‐F_c_ map does reveal continuous NSL density that could be explained by the keto form of the HPP occupying the position that it has in the 100 K complex in the uninhibited active sites (Figure 5b). However, the limited resolution does not permit us to model a second HPP molecule in this active site.

**FIGURE 6:**
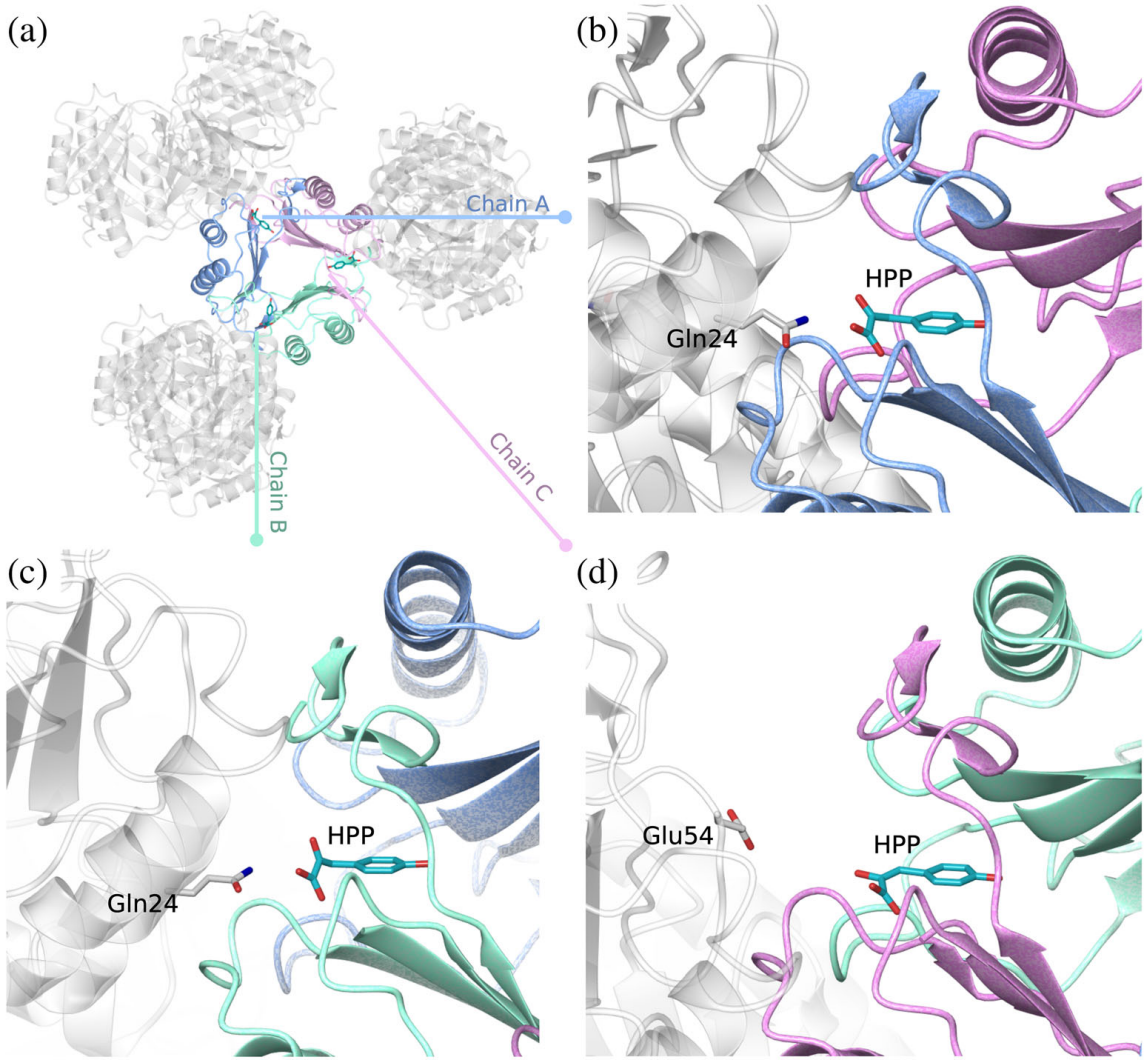
Crystallographic contacts of the MIF trimer. (a) The MIF trimer (colored chains) interacts with symmetry related subunits (gray) within the P2_1_2_1_2_1_ crystal lattice. (b, c) Active sites of chains A and B are partially occluded by crystal contacts including symmetry‐related residues such as Gln‐24 restricting solvent access. (d) The active site of chain C is more solvent accessible due to a lack of encroaching crystal contacts, with the nearest symmetry related residue, Glu‐54, facing away from the active site.

Active sites A and B exhibit reduced solvent accessibility when compared to chain C due to occlusion from crystal lattice packing (Figure 6). In chains A and B, crystal contacts with neighboring symmetry‐related molecules partially bury the active sites. This is evident in active sites A and B where the neighboring symmetry‐related residues adopt conformations that restrict the active site, limiting solvent accessibility (Figure 6b,c). In contrast, the chain C active site remains largely solvent exposed with no residue from a symmetry‐related chain encroaching upon the active site (Figure 6d). In active site C, the carboxyl group of the HPPenol also forms an electrostatic interaction with Lys‐32 as observed in chains A and B. Similarly, the HPPenol carboxyl is positioned between the Lys‐32 side chain amino group and the Pro‐1 nitrogen, with its oxygen atoms less than 2.6 Å from the nitrogen atoms of either of these groups supporting hydrogen bond interactions as in chains A and B. As observed for the HPP bound to the other two active sites, the aromatic ring of the HPPenol occupies the center of the active site, with the edge of its ring facing the π‐face of the aromatic ring of Tyr‐95 of chain B. The phenol hydroxyl group of HPPenol donates a hydrogen bond to the side chain oxygen of Asn‐97 of chain B.

### 2.3 Catalytic implications of the neutron structure

**FIGURE 7:**
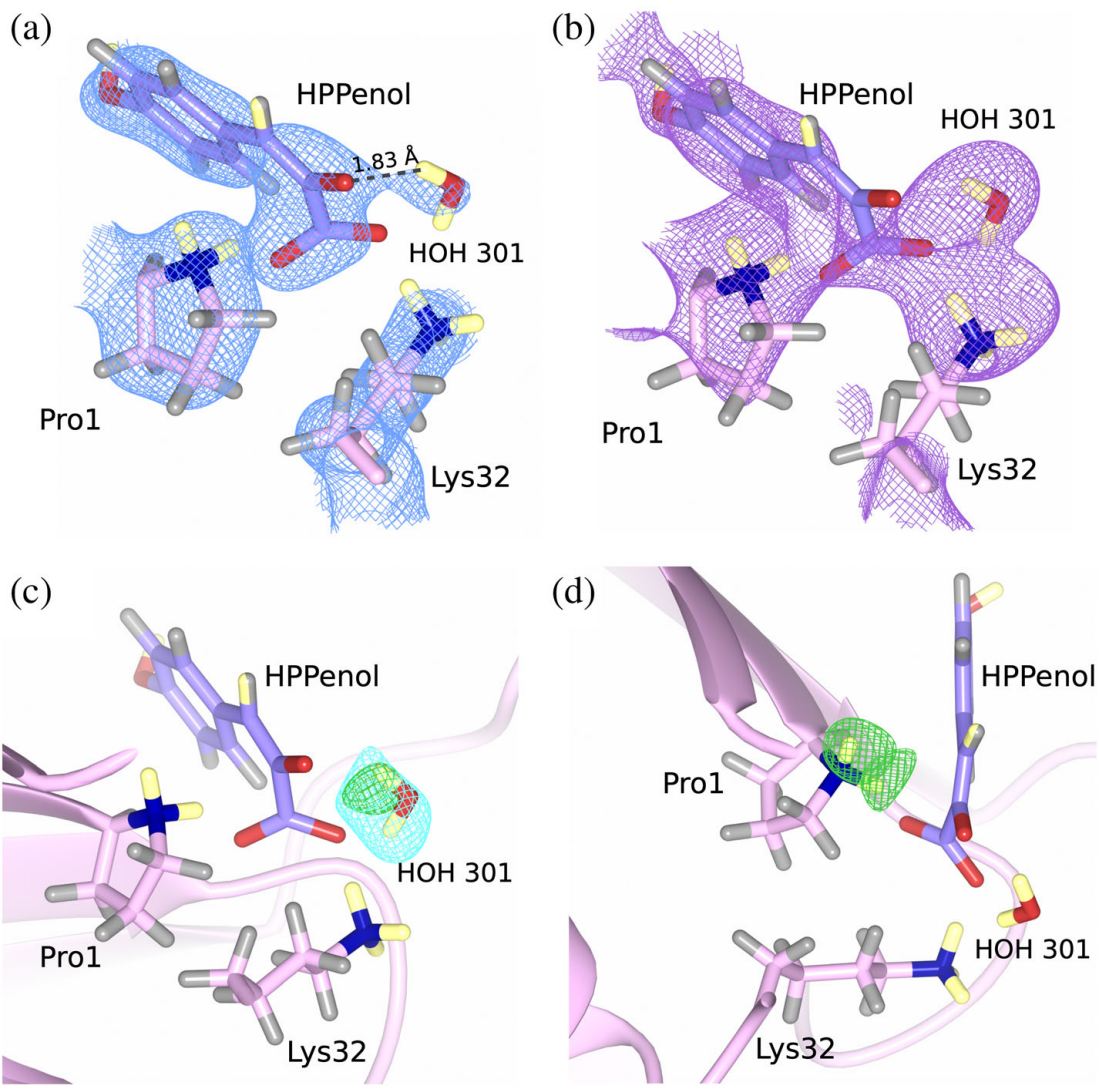
Positioning of key water molecule near the enolate. (a) 2F_o_‐F_c_ electron density (blue) and (b) NSL density (purple) maps contoured at 0.90σ and 1.0σ, respectively, showing the proximity of a water molecule HOH C 301 to the enolate oxygen of HPPenol with deuterium atoms in yellow and hydrogen atoms in gray. (c) F_o_‐F_c_ NSL omit map (green) contoured at 3.0σ, in which the HOH C 301 water deuterium atom closest to the enolate group was omitted from the map calculation and an F_o_‐F_c_ electron density omit map (aquamarine) in which the HOH C 301 water was omitted. (d) F_o_‐F_c_ NSL omit map (green) contoured at 3.30σ, in which the Pro1 D2 and D3 deuterium atoms have been omitted, confirming its doubly protonated state.

The unique environment of the solvent‐exposed active site of chain C provides insight into the catalytic tautomerization mechanism of MIF. Notably, active site C is the only active site in which the product form, HPPenol, is the predominant species. Of catalytic significance in active site C is the presence of a nearby water molecule, HOH C 301, positioned 1.83 Å from the O4 atom of HPPenol (Figure 7a). This proximity supports alternative modeling in the neutron structure in which the HPPenol is deprotonated at O4, forming a negatively charged enolate ion with the deuteron bound to HOH C 301 instead of the HPP O4 atom (Figure 7b).

To support this, an NSL F_o_‐F_c_ density map calculated with the negatively charged enolate modeled, but omitting the HOH C 301 deuterium atom facing HPPenol, reveals a difference peak suggesting that the deuterium is covalently bound to the HOH C 301 oxygen and not to the enolate O4 oxygen. An electron density omit F_o_‐F_c_ map of the HOH C 301 molecule further confirms the position and presence of the water molecule in this position (Figure 7c). We are likely capturing an average of both states: the neutral enol and deprotonated enolate intermediate present prior to abstracting a deuterium from the water molecule to complete the tautomerase reaction. However, these observations indicate that the deprotonated enolate form is evidently the predominant species in the crystal. The NSLD omit F_o_‐F_c_ maps also confirm that Pro‐1 is doubly deuterated, likely following a hydrogen atom abstraction event from the C3 atom of HPP (Figure 7d). Thus, we have snapshots of both the beginning and end of the tautomerase reaction. In the initial state, HPP is bound in the keto form with a singly protonated Pro‐1, positioned to abstract a hydrogen atom from the HPP C3 carbon. Formation of the enolate is initiated by hydrogen atom abstraction by Pro‐1, resulting in a doubly protonated Pro‐1 residue. Subsequent HPP C2‐C3 double bond formation and displacement of the C2 carbonyl π‐electrons result in formation of a negatively charged O4 atom which is poised for protonation by HOH C 301 to complete the keto to enol conversion. The beginning of this process was also captured in our earlier structure (PDB ID 3IJJ) with Pro‐1 proximal to carbon C3 of the keto form, positioned for H atom abstraction. The final stage of the reaction is observed in the joint XN‐refined structure reported here, with a water molecule donating a hydrogen atom to the enolate oxygen to produce the final enol product.

### 2.4 Position of HPP C3 atom in an ambient temperature x‐ray study

**FIGURE 8:**
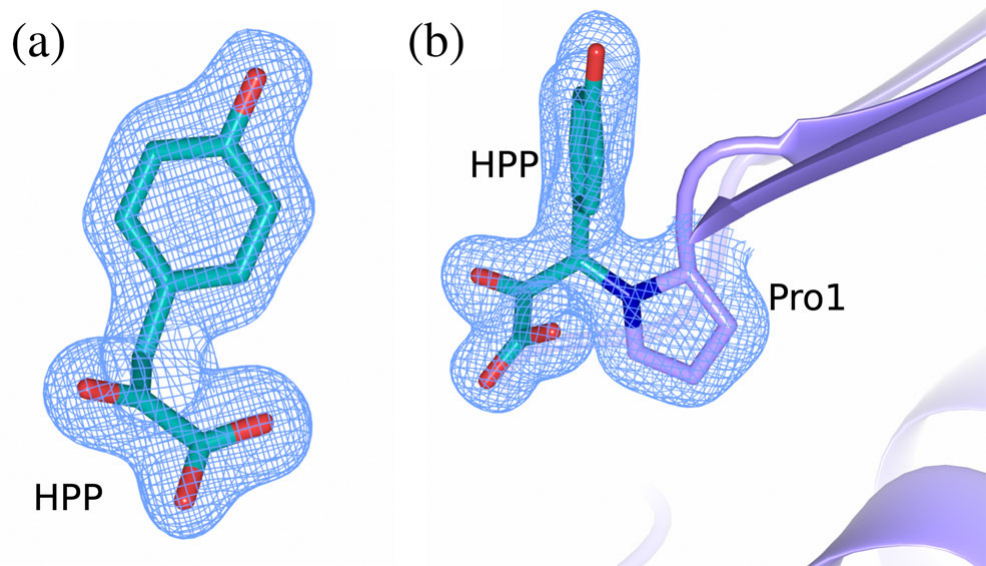
Covalent adduct formed in a room‐temperature synchrotron x‐ray crystallographic structure of MIF‐HPP induced by radiation damage (PDB ID 8TT9). (a) 2F_o_‐F_c_ electron density (blue) map of HPP in the room temperature crystal structure contoured at 1.0σ. (b) Covalent bond between the HPP and the Pro‐1 nitrogen with electron density contoured at 1.0σ.

A room‐temperature synchrotron x‐ray data set was collected for this complex. Interestingly, the electron density indicates a covalent bond between atom C3 of the HPP and the nitrogen of Pro‐1 (Figure 8). This covalent adduct not only differs from the room‐temperature rotating anode x‐ray and neutron results and the cryogenic synchrotron results, but is also inconsistent with the reversible steady‐state kinetics of the tautomerase reaction of MIF. Thus, this observation is likely an artifact due to radiation damage of the sample, which occurs readily in room‐temperature x‐ray diffraction experiments, but is reduced at cryogenic temperatures due to immobility of the radiation‐induced radicals generated. The ability of these radicals to diffuse throughout the sample and react with the protein and induce damage is limited at cryogenic temperatures (Ravelli & McSweeney, 2000). HPP has been reported to serve as an antioxidant that is oxidized as it scavenges free radicals (Scrima et al., 2017). Such antioxidants protect proteins from oxidation (Gulcin, 2020). It has been reported that, under conditions that generate radicals, alpha‐keto acids are more reactive in their enol tautomer, preferentially forming enol radicals (Schmittel & Rock, 1992). According to Schmittel and Röck, this cationic radical forms at the carbon directly bonded to the carbon of the enol group and is resolved readily by reaction with an amine that attacks this carbon by a nucleophilic mechanism, resulting in a keto product covalently bound to the amine (Schmittel & Rock, 1992). In HPP, this reactive carbon corresponds to the C3 carbon. In the MIF structure studies at room temperature, exposure to synchrotron radiation evidently results in generation of the radical cation of HPPenol at carbon C3 that is proximal to Pro‐1. This radical located on C3 ultimately reacts with the Pro‐1 nitrogen, forming the MIF‐HPP (keto) covalent adduct seen in the electron density map (Figure 8). Although artifactual, this interaction demonstrates the proximity of the C3 atom to the active site proline in a well‐defined 1.68 Å resolution structure. This strengthens the neutron diffraction results reported here, as it verifies the geometry proposed in the initial step of the deduced catalytic mechanism.

## 3 DISCUSSION

The neutron diffraction data presented here provide detailed insight into the MIF tautomerase reaction by visualizing the position of hydrogen atoms in the active site. In the reported structure, the HPP substrate is bound in all three chains of the MIF trimer. The amino group of Lys‐32 is triply deuterated, and thus in the expected positively charged state in all three active sites. Lys‐32 mediates an electrostatic interaction with the carboxyl group of HPP, which anchors the substrate along with hydrophobic interactions and hydrogen bonding to Asn‐97.

Of particular note is the proximity of a key water molecule near the HPPenol product. The solvent content of the crystal used for the x‐ray/neutron structure is approximately 56%. As with protein crystals in general, water flows through solvent channels in the crystal. The structure that is determined represents an average structure over the course of time of data collection and averaged over all of the asymmetric units within the crystal lattice. Most of the water molecules are not seen in the structure because they are averaged‐out in the resulting maps due to their mobility. Any water molecules that are observed, therefore, are held in place by some interaction with the protein or with another molecule held in that position by interactions with the protein. HOH C 301, besides its close hydrogen bond with the enolate oxygen of HPPenol, also donates a hydrogen bond to atom O1 of the HPPenol carboxyl group. The HPPenol carboxyl group, in turn, forms a salt bridge with the side chain of Lys‐32 of chain C. The position of the HPPenol is further stabilized by the other interactions mentioned in the Results section (i.e., with Tyr‐95 and Asn‐97 of chain B). Thus, the anchoring of HPPenol by the protein stabilizes the position of the water molecule that interacts with HPPenol, as demonstrated by the visibility of the water molecule in both the NSL and electron density maps. The orientation of this water molecule, with a hydrogen atom that forms a 107.18° angle around the enolate oxyanion only 1.83 Å away, suggests its role in donating H^+^ to the enolate.

The fact that the enolate is in the anionic form and the proximal water molecule has both hydrogens associated indicates that the relative pKa values of water and the enol product within the active site are such that the equilibrium is shifted toward the product being deprotonated. The pKa has been measured for an acetone enol to be approximately 10.9 (Chiang et al., 1984), whereas water has a pKa of 15.7. However, the proximity of a carboxylate, its ionic interaction with the protein, and the protein active site environment would likely change this value for HPPenol. The unprotonated enolate observed in this structure would indicate that this pKa is less than that of water.

**FIGURE 9:**
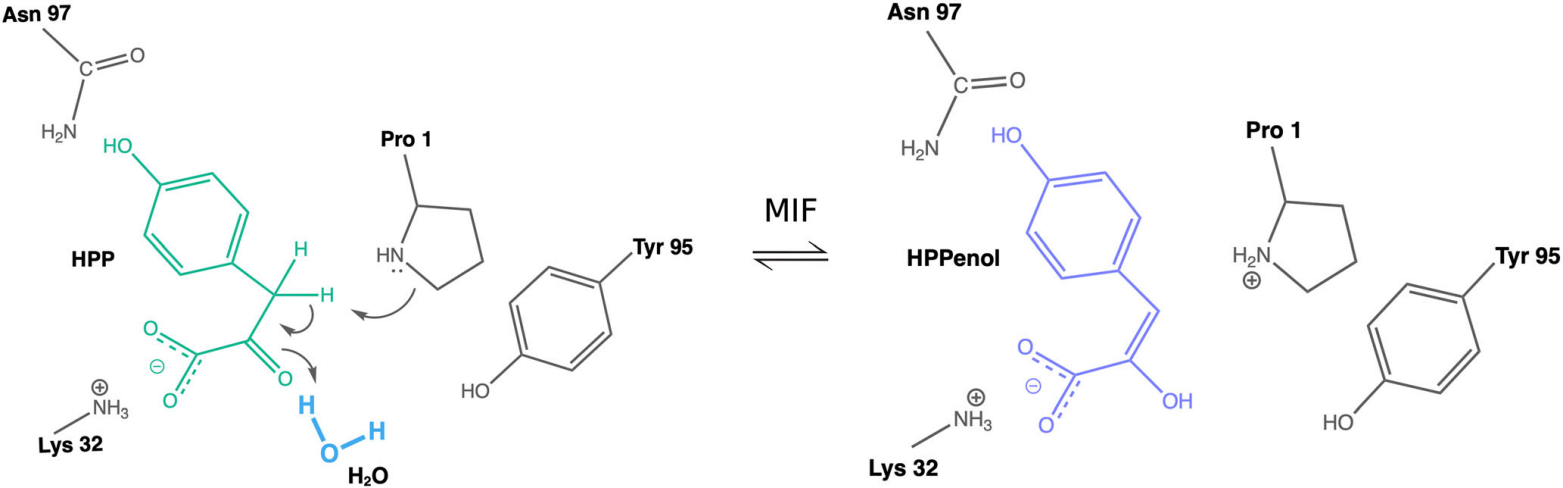
Proposed reaction mechanism of MIF. The MIF reaction is initiated with HPP C3 atom hydrogen abstraction by the N‐terminal Pro‐1 residue. This generates an enolate intermediate that is converted to the enol product by abstraction of a proton from a key nearby water molecule, as elucidated by the current neutron protein crystallography study.

The joint x‐ray/neutron structure reported here provides insight into the mechanism of MIF catalysis (Figure 9). To initiate the keto to enol tautomerase reaction, hydrogen must be abstracted from the substrate. This necessitates that the C3 atom of the keto form is proximal to the Pro‐1 nitrogen when it binds. This is observed in our previous study (Cho et al., 2010) and also indicated by the NSLD maps presented herein. Our model indicates that the reaction is initiated by hydrogen abstraction by the Pro‐1 nitrogen, as was earlier proposed (Lubetsky et al., 1999), resulting in the formation of a doubly protonated Pro‐1 residue. HPP is converted into an enolate form, and to complete the tautomerase reaction, a hydrogen ion must be added to the O4 oxyanion of the enolate that is formed after abstraction of a hydrogen atom from carbon C3. Our structure confirms the presence of a proton donor, HOH C 301, which is in a prime position to donate a proton/deuteron to the O4 atom of the enolate, generating the enol product. It has been proposed in a study using murine MIF that Pro‐1 possibly is the source that donates the proton to the O4 atom (Stamps et al., 2000). However, our results with human MIF indicate that this is actually a water molecule that donates the proton to the enolate oxygen.

The structural evidence presented here provides a comprehensive picture of the MIF tautomerase mechanism in which the Pro‐1 nitrogen abstracts a proton from C3 of the keto form of HPP to generate an enolate intermediate which is converted to the enol product by abstraction of a proton from a nearby water molecule (Figure 9). The detailed information of proton positioning resolved here with neutron protein crystallography provides insight into previously unresolved questions regarding the mechanistic details of MIF, a potential disease target that possesses diverse physiological functions.

## 4 MATERIALS AND METHODS

### 4.1 Protein purification

Human MIF was purified in a manner similar to that described by Sun et al., 1996 (Sun et al., 1996). Specifically, human MIF was expressed in BL21(DE3) cells, which were lysed in 20 mM tris(hydroxymethyl)aminomethane (Tris), pH 7.4, 20 mM sodium chloride. The protein was purified by ion exchange chromatography, using an anion‐exchange column connected in series to a cation‐exchange column at pH 7.4. The MIF was collected in flow‐through fractions with most impurities binding to one or the other ion exchange column. This provided protein that was at least 95% pure as judged visually by Coomassie staining of SDS‐polyacrylamide gels. A size‐exclusion chromatography step was afterwards employed to purify the protein further. This resulted in essentially 100% pure protein as judged by Coomassie staining.

### 4.2 Deuteration of substrate and crystallization

For room temperature synchrotron data collection, crystals were grown in a similar fashion to that previously described (Crichlow et al., 2009), mounted in quartz capillaries with plugs of crystallization solution to maintain crystal hydration and shipped.

For room temperature neutron data collection, MIF was co‐crystallized with HPP deuterated at carbon C3 (^2^H‐HPP). Deuterated HPP was made by preparing a 100 mM stock of HPP in 0.5 M ammonium acetate pD 8.5 in D_2_O and allowing this to incubate for 3 days at room temperature, based on a similar protocol used by Rétey et al. (1977). Sitting drops were set up by mixing 5–30 μL of 1 mM MIF, 10 mM ^2^H‐HPP, 20 mM NaCl in 20 mM Tris (pH 7.4) mixed with an equal volume of reservoir solution containing 2 M (NH_4_)_2_SO_4_, 1% isopropanol in 100 mM Tris (pH 7.4) at 22°C. Two weeks prior to neutron data collection, the largest crystals (~0.3 mm^3^) were mounted in quartz capillaries for room temperature data collection. Plugs of crystallization solution were prepared with D_2_O to exchange solvent molecules and labile hydrogen atoms of the protein through vapor exchange.

### 4.3 Neutron and x‐ray data collection and MIF‐HPP XN structure refinement

Quasi‐Laue neutron diffraction data were collected at room temperature on the IMAGINE instrument at the High Flux Isotope Reactor (HFIR) (Meilleur et al., 2013; Meilleur et al., 2020; Schröder et al., 2018) at Oak Ridge National Laboratory. An incident neutron wavelength range of 2.8–4.5 Å was used. A total of 24 diffraction images were collected in three unique crystal orientations with an exposure time of 35 h per frame and a Δφ of 10° between frames for each orientation.

Following neutron diffraction data collection, an x‐ray dataset was collected on the same crystal at room temperature on a home‐source x‐ray diffractometer.

The quasi‐Laue neutron data were indexed and integrated using *LAUEGEN* prior to wavelength normalization and scaling with *LSCALE* (Arzt et al., 1996; Campbell et al., 1998; Helliwell et al., 1989) and *Scala* (Evans, 2006; Winn et al., 2011). The x‐ray data were indexed and integrated using *CrysAlis PRO* (Rigaku, Woodlands, Texas, USA) and scaled and merged with *AIMLESS* in the *CCP4* suite (Evans & Murshudov, 2013). The data reduction statistics are presented in Table 1.

**TABLE 1:** Data collection and refinement statistics of the joint x‐ray/neutron‐refined structure and of the room‐temperature crystal structure from synchrotron x‐ray data.

	Joint x‐ray/neutron (PDB ID 8TT8)		Room‐temperature synchrotron (PDB ID 8TT9)
	X‐ray	Neutron	
Wavelength (Å)	1.54	2.80–4.50	1.00
Resolution range (Å)	54.69–1.75 (1.78–1.75)	17.62–2.5 (2.59–2.5)	44.21–1.68 (1.71–1.68)
Space group	P 21 21 21		P 21 21 21
Unit cell (Å, °)	68.61 69.23 89.18, 90 90 90		68.57 68.87 88.43, 90 90 90
Total reflections	204,248 (10809)	69,080 (3314)	370,027 (15859)
Unique reflections	43,601 (2366)	11,735 (964)	48,464 (2461)
Multiplicity	4.7 (4.6)	5.9 (3.4)	7.6 (6.4)
Completeness (%)	100.00 (100.00)	80.00 (66.60)	100.0 (100.0)
Mean I/sigma(I)	10.4 (2.6)	5.70 (2.20)	16.1 (0.7)
R‐merge	0.086 (0.586)	0.2510 (0.3490)	0.076 (2.208)
R‐meas	0.101 (0.649)	0.2690 (0.3980)	0.085 (2.426)
R‐pim	0.044 (0.310)	0.0920 (0.1810)	0.040 (1.302)
CC1/2	0.988 (0.770)	0.948 (0.679)	0.998 (0.314)
Reflections used in refinement	43,541	11,721	48,298
Reflections used for R‐free	2136	579	2346
R‐work	0.1540	0.2189	0.1703
R‐free	0.1805	0.2705	0.1992
RMSD (bonds) (Å)	0.017		0.0144
RMSD (angles) (°)	1.39		1.3355
Ramachandran favored (%)	98.50		98
Ramachandran allowed (%)	1.50		2
Ramachandran outliers (%)	0.00		0
Rotamer outliers (%)	1.93		1
Clashscore	3.35		4.76
Average B‐factor (Å^**2**^)	23.79		33.87
Macromolecules	22.17		32.80
Ligands	32.95		44.01
Solvent	43.50		48.25

*Note*: Statistics for the highest‐resolution shell are shown in parentheses.

The 100 K x‐ray structure (PDB ID 3IJJ) was used as the starting model for refinement against the room temperature x‐ray data. Water molecules and HPP atoms were stripped from the initial model. Rigid body refinement was first performed followed by positional and individual B‐factor refinement using *phenix.refine* in the *Phenix* suite (Liebschner et al., 2019). Water molecules and HPP atoms were built manually in *Coot* (Emsley et al., 2010). Hydrogen atoms were added to non‐exchangeable positions to the final room temperature x‐ray model using *Phenix.ready_set* and hydrogen and deuterium atoms were added to exchangeable positions. This model was used to perform joint refinement against both the x‐ray and the neutron datasets (XN refinement). Positional, B‐factor and occupancy refinement was performed using *phenix.refine*. Manual optimization of the model was done in *Coot* (Emsley et al., 2010). The statistics of the XN MIF‐HPP structure are presented in Table 1. Figures displaying structural results were made using CCP4mg (McNicholas et al., 2011). Figure 4a was made using PoseEdit (Diedrich et al., 2023).

### 4.4 Synchrotron data collection and room temperature MIF‐HPP x‐ray structure

A synchrotron x‐ray dataset was collected at room temperature on ALS beamline 4.2.2. The data reduction statistics are presented in Table 1. The 100 K x‐ray structure (PDB ID 3IJJ) was used for the phases for the room temperature synchrotron x‐ray data. Water molecules and HPP atoms were stripped from the initial model. Rigid body refinement was first performed followed by positional and individual B‐factor refinement using *phenix.refine* in the *Phenix suite* (Liebschner et al., 2019). Water molecules and HPP atoms were built manually in *Coot* (Emsley et al., 2010). The refinement statistics of the room temperature x‐ray MIF‐HPP structure are presented in Table 1.

## AUTHOR CONTRIBUTIONS

**Elias J. Lolis:** Writing – original draft; writing – review and editing; formal analysis; supervision. **Georgios Pantouris:** Investigation; writing – review and editing. **Ewelina Jablonowski:** Investigation; writing – review and editing. **Gregg V. Crichlow:** Conceptualization; investigation; writing – original draft; writing – review and editing; formal analysis. **Gabriela C. Schröder:** Investigation; writing – original draft; writing – review and editing; formal analysis. **Jay Nix:** Investigation; writing – review and editing; formal analysis. **Flora Meilleur:** Investigation; writing – original draft; resources; methodology; writing – review and editing; formal analysis; supervision. **Naomi Chayen:** Writing – review and editing; methodology.

## Data Availability

The data that support the findings of this study are openly available in the Protein Data Bank at https://www.rcsb.org, accession IDs 8TT8 and 8TT9.
